# The Effect of Diabetes Management Shared Care Clinic on Glycated Hemoglobin A1c Compliance and Self-Management Abilities in Patients with Type 2 Diabetes Mellitus

**DOI:** 10.1155/2023/2493634

**Published:** 2023-12-31

**Authors:** Tian Jiang, Chao Liu, Ping Jiang, Wenjun Cheng, Xiaohong Sun, Jing Yuan, Qiaoling Wang, Yanlei Wang, Shihui Hong, Haiyan Shen, Dongchun Zhu, Yi Zhang, Fang Dai, Jing Hang, Jiguo Li, Honglin Hu, Qiu Zhang

**Affiliations:** ^1^Department of Endocrinology, The First Affiliated Hospital of Anhui Medical University, Hefei 230022, Anhui, China; ^2^Department of Outpatient Changjiang Road, The First Affiliated Hospital of Anhui Medical University, Hefei 230061, Anhui, China; ^3^Department of Pharmacy, The First Affiliated Hospital of Anhui Medical University, Hefei 230022, Anhui, China; ^4^Beijing Health Technology Co., LTD, Beijing 100085, China

## Abstract

**Objective:**

We aim to evaluate the impact of diabetes management shared care clinic (DMSCC) on glycated hemoglobin A1c (HbA1c) compliance and self-management abilities in patients with type 2 diabetes mellitus (T2DM).

**Methods:**

This study was a prospective cohort study of patients with T2DM participating in the DMSCC. At baseline and after management, the HbA1c levels were measured, the HbA1c compliance rate were calculated, and the Summary of Diabetes Self-Care Activities-6 (SDSCA-6), Diabetes Empowerment Scale-DAWN Short Form (DES-DSF), and Problem Areas in Diabetes Scale—Five-item Short Form (PAID-5) were completed. These pre- and post-management data were compared.

**Results:**

A total of 124 eligible patients were enrolled. After the diabetes management of DMSCC, the average HbA1c decreased and the HbA1c compliance rate increased significantly (*P* < 0.01). SDSCA-6 showed significant improvement in physical activity, glycemic monitoring, smoking (*P* < 0.01), and taking medication (*P* < 0.05). DES-DSF suggested a greater willingness to try to effectively treat diabetes (*P* < 0.05). PAID-5 indicated significant improvement in diabetes-related emotional distress.

**Conclusion:**

DMSCC can help patients with T2DM reduce HbA1c, increase HbA1c compliance, improve diabetes self-management behaviors, empowerment, and diabetes-related emotional distress and serve as an effective exploration and practice of diabetes self-management education and support.

## 1. Introduction

Diabetes mellitus has become one of the most common and serious chronic progressive diseases in current society. The prevalence of diabetes in adults worldwide already exceeds 10.5% and is projected to grow to 12.2% by 2045, the majority of which is type 2 diabetes mellitus (T2DM) [1]. The current severe situation is not only the high prevalence rate but also a low awareness rate, a low treatment rate, and a low compliance rate [2, 3]. Among T2DM patients in China, less than 40% achieved the target of 7% of glycated hemoglobin A1c (HbA1c), and only 9.2%–16.7% were able to adequately engage in diabetes self-management behaviors [4]. In order to cope with the increasing prevalence and poor glycemic control, reduce or delay the occurrence of diabetes complications, the treatment of diabetes cannot be limited to drug intervention, but needs to be systematically, comprehensively, and continuously managed. Furthermore, it is necessary and ongoing to update and change diabetes treatment and management concepts. 

Shared care has been used in a variety of chronic disease management services [5–11], such as asthma, chronic obstructive pulmonary disease (COPD), pulmonary arterial hypertension (PAH), hypertension, mental illness, and maternal management. The concept of shared care applied to diabetes management originated in the United Kingdom [12] and was subsequently applied and developed in the United States, China, Australia, and other countries [13–19]. In the past decades, the shared care model has been tried to apply to patients with type 2 diabetes, focusing on combining the disease-specific expertise of endocrinologists with the general health and daily life knowledge of general practitioners in order to provide qualified medical services [20]. However, despite developments in the field of self-management education and psychosocial care during the past decade, too many patients with diabetes receive inadequate care and support to enable them to achieve optimal health and well-being [21].

Shared care clinic is one of the further innovative approaches in the exploration of diabetes education and management, its essence is the integration of multidisciplinary teams for effective continuous management of patients with diabetes. Most of the previous shared care models for diabetes were all carried out in primary practice, while others used to carry out daily visits in primary health care and annual examinations in specialist clinics [20]. In fact, our diabetes management shared care clinic (DMSCC) is also a combination of primary health care and specialized health care. Multidisciplinary professional doctors provide treatment guidance and adjustment during the annual check-up at least 2–4 times a year. At other times, online caregivers provide follow-up, guidance, and reminders, which are similar to those of primary health care, but their functions are more comprehensive and timely than those of the basic primary health care.

2017 National Standards for Diabetes Self-Management [22] published by the American Diabetes Association (ADA) and the American Association of Diabetes Educators (AADE) recognize diabetes self-management education and support (DSMES) as a critical element of care for all people with diabetes and those at risk for developing the condition. This is the first time to combine diabetes self-management education (DSME) and diabetes self-management support (DSMS), that means education and support are equally important, reflecting the value of ongoing support and multiple levels of service. The DMSCC happens to be the carrier for realizing multidisciplinary continuous diabetes education, support, and management.

The DMSCC provides systematic medical guidance inside and outside the hospital around the seven aspects of taking medication, monitoring, healthy eating, being active, problem solving, healthy coping, and reducing risk. This concept originates from American Association of Diabetes Educators-7 (AADE-7) Self-Care Behaviors® [23]. AADE-7 is a robust framework for self-management of diabetes to achieve behavioral change and serves as the cornerstone of DSMES to guide healthcare teams in effective person-centered collaboration and goal setting to achieve health-related outcomes and improve quality of life [23]. Research shows that patients with T2DM participating in DSMES can reduce HbA1c by up to 1% [24]. Shared care in the early stages has also been confirmed to help patients improve blood sugar, weight, and blood pressure; achieve higher engagement and compliance; and is recognized by both doctors and patients [19]. As a further exploration method to realize DSMES, DMSCC is still lacking in research works on its effects on the level of HbA1c and diabetes self-management abilities of patients with T2DM. The purpose of this study is to assess changes in HbA1c compliance, self-management behaviors, empowerment ability, and diabetes-related emotional distress in patients with T2DM before and after the intervention of the diabetes management shared care clinic, and obtain authentic localization evidence for assisted analysis.

## 2. Methods

### 2.1. Study Design and Settings

This study is a prospective cohort study based on the management of patients with T2DM participating in the DMSCC to assess the impact of DMSCC on blood glucose status and self-management abilities. Among the diabetes patients treated by the endocrine department of the First Affiliated Hospital of Anhui Medical University, no matter they are diagnosed or newly diagnosed in the community hospital or specialized hospital, as long as they have mild to moderate hyperglycemia and no serious complications of diabetes or other serious diseases, they are eligible to participate in DMSCC.

If the patient met the criteria and participated in this study, the types of hypoglycemic drugs used by the patients generally remained unchanged during the study period. Unless hypoglycemia or HbA1c exceeded the control standard by more than 1%, our management team would analyze the causes of hypoglycemia or hyperglycemia and adjust the drug doses appropriately if necessary.

At baseline and after management of DMSCC, the change of HbA1c of the included patients was observed. At the time of the first visit and the return visit after management, the scales (Chinese version) were completed face-to-face to assess the change of their diabetes self-management abilities. Patients can fill in the scales independently and ask the doctors if they have questions. Doctors can explain the problem items but should not guide or interfere with the patient's choices.

This study and article were approved by the Medical Ethics Committee of the First Affiliated Hospital of Anhui Medical University (Quick-PJ2022-11-27).

### 2.2. Inclusion Criteria and Exclusion Criteria

The inclusion criteria that can be included in this study were (1) aged 18–80 years old, (2) conformed to the 1999 World Health Organization (WHO) diagnostic criteria for T2DM, (3) voluntary and cooperative participation in the management of DMSCC and this study, (4) to have at least one follow-up visit after participating in the management and to complete the assessment of diabetes self-management ability-related scales before and after the management, and (5) signed informed consent for data and services related to the shared care model.

Exclusion criteria: (1) unconscious, unable to cooperate, and unclear language expression and (2) patients with severe diabetes complications, such as diabetes nephropathy stage IV and above, diabetes retinopathy stage IV and above, and diabetes foot.

### 2.3. The Process of Diabetes Management Shared Care Clinic

The DMSCC of the Endocrinology Department of the First Affiliated Hospital of Anhui Medical University is divided into in-hospital management and out-of-hospital management. In-hospital management is carried out in the outpatient consulting rooms of the Endocrinology Department. The endocrinologists, diabetes specialist nurses, nutritionists, sports instructors, pharmacists, etc., communicate with patients individually and write prescriptions, respectively. Different roles can optimize or adjust the prescription after discussing key issues or different opinions to provide more reasonable multidimensional medical advice. The out-of-hospital management is conducted online, and the online caregivers provide continuous services. DMSCC takes patients as the center to carry out integrated management and continuous comprehensive care inside and outside the hospital, encourage self-management and support to help patients improve metabolic indicators, enhance self-management capabilities, and obtain certain social support.

The in-hospital process is data establishment, sign-in, basic physical examination, scale evaluation, doctor consultation, patient education, and then leaving the clinic. In the patient education session, diabetes specialist nurses, nutritionists, sports instructors, and pharmacists individually assessed and guided diabetes care, nutrition, exercise, and medication management in detail, and record them systematically. Our hospital characteristically adds the role of pharmacist to the management team to provide more professional medication education and improve medication compliance. During follow-up visits, the team adjusted the treatment plan and related care and guidance content according to the patient's condition.

Then, out-of-hospital management is realized through the APP “Shared Care” (Chinese version, Version 2.0.2, iHealth Labs China Co., LTD.). The out-of-hospital management process includes providing management plans, reminding blood sugar monitoring, warning intervention for blood sugar levels exceeding standards, weekly measurement reports, reminding to take medicines, online Q&A, online follow-up, knowledge push, and reminding in-hospital follow-up time. The trained online caregivers from Beijing iHealth Technology Co., LTD. can track the changes or emerging problems of the patients on the APP and recorded and dealt with 7 aspects [23] of taking medication, monitoring, diet, exercise, problem solving, healthy coping, and reducing risk through a SOAP (subjective-objective-assessment-plan) approach, so that the shared care mode has continuity inside and outside the hospital. These online caregivers basically have nurse qualification certificates and nurse practice certificates certified by the National Health Commission of the People's Republic of China (PRC). In addition, they may also have a licensed pharmacist qualification certificate certified by the National Medical Products Administration, a public dietitian certificate certified by the National Health Commission of PRC, a registered nutrition technician certificate certified by the Chinese Nutrition Society, or a health manager certificate certified by the Ministry of Human Resources and Social Security of PRC, etc. Therefore, they have the ability to receive specialized medical training and provide online guidance in different aspects of diabetes management.

### 2.4. Data Collection

At baseline, demographic and clinical data of T2DM patients enrolled in this study were recorded, including age, gender, duration of diabetes, weight, height, body mass index (BMI), systolic blood pressure (SBP), diastolic blood pressure (DBP), and HbA1c. BMI was calculated as the weight (kg) divided by the square of the height (m). Blood pressure was measured with a standard mercury sphygmomanometer after sitting for at least 10 minutes, and the average value of two measurements is obtained. Venous blood was extracted to measure the levels of HbA1c. HbA1c was detected by high performance liquid chromatography (HPLC).

Furthermore, patients completed baseline scales according to their current actual status, which included Summary of Diabetes Self-Care Activities-6 (SDSCA-6), Diabetes Empowerment Scale-Diabetes Attitudes, Wishes, and Needs Short Form (DES-DSF), and Problem Areas in Diabetes Scale—Five-item Short Form (PAID-5).

After guidance and follow-up in the DMSCC, the patients were retested for HbA1c and the above scales to evaluate their self-management abilities after receiving diabetes management knowledge in the shared care clinic.

Among the outcome variables, the primary outcomes were the levels of HbA1c, HbA1c compliance rate, HbA1c adverse rate, and the results of SDSCA-6, while the secondary outcomes were the results of DES-DSF and PAID-5.

### 2.5. Self-Management Ability Assessment–Related Scales

Summary of Diabetes Self-Care Activities-6 (SDSCA-6) is a brief self-report questionnaire used to assess self-management behaviors of patients with T2DM. The scale contains 7 items, which are used to evaluate the daily life behaviors of patients in 6 dimensions of diet, exercise, blood glucose monitoring, foot care, drug use, and smoking. In addition to the question of whether to smoke, the other 6 scores indicate the number of days that the patient has adhered to the behavior in the past 7 days. The options are from 0 days to 7 days, and the number of days is the corresponding score. The maximum score for each item is 7 points, with higher scores indicating better self-management. It is recommended to be used at the initial patient visit to assess behavioral status in the absence of diabetes education, and to conduct periodic assessments after receiving diabetes education, aiming to observe the impact of diabetes education on patients' self-management behavior changes [21, 25].

Diabetes Empowerment Scale-DAWN Short Form (DES-DSF) is a subscale from a series of scales developed by a multinational study called the Diabetes Attitude, Wishes, and Needs Study (DAWN). There are 5 items in this scale, each item has 5 options, assigned a scale of 1 to 5, ranging from “never” to “always.” The total score is the sum of the 5 item scores multiplied by 4 (range: 20–100 points). Higher scores indicate stronger empowerment of patients in diabetes self-management, whereas lower scores indicate poorer empowerment. The questionnaires were translated into the major local languages of 17 countries, followed by a back-translation and coordination process to ensure consistency with the original questionnaires. The Cronbach *α* of the Chinese version of DES-DSF is 0.68 [26, 27].

Problem Areas in Diabetes Scale—Five-item Short Form (PAID-5) mainly measures the psychological distress of patients with diabetes, including the assessment of fear, depression, and the needs of patients with diabetes. The sensitivity and specificity of PAID-5 are reliable. Patients choose the severity of their diabetes problems according to their own personal feelings. PAID-5 is single dimensional with a total of 5 items, and the scale adopts the Likert 5-level scoring method. Higher scores indicate greater diabetes-related psychological distress [28, 29].

### 2.6. Statistics Analysis

SAS version 9.4 for Windows (SAS Institute Inc., Cary, NC, USA) was used for statistical analysis of the data, and the Shapiro–Wilk (Shapiro–Wilk normality, S–W) test was used to test the normality of measurement data. The quantitative data that completely or approximately conformed to the normal distribution were expressed as $\bar{x}$ ± *s*, and the paired-sample *t*-test was used for comparison before and after the management of the DMSCC. The quantitative data with skewed distribution were expressed as median (interquartile range), and the paired-sample Wilcoxon signed-rank test was used for comparison before and after management of the DMSCC. Qualitative data were expressed as proportions (%), and a paired chi-square test (McNemar's test) was used for comparison before and after management of the DMSCC. A value of *P* < 0.05 was considered statistically significant in the statistical tests.

## 3. Results

### 3.1. Patient Characteristics

From April 2020 to April 2022, a total of 1724 T2DM patients received basic diabetes care, of which 124 eligible patients participated in this study and accepted the management of DMSCC.

At baseline, according to the inclusion and exclusion criteria, a total of 124 patients with T2DM were finally included in this study. Among them, there were 76 males (61.29%) and 48 females (38.51%), with a mean age of 54.57 ± 12.23 years and a mean duration of T2DM of 6.85 ± 7.21 years. At baseline, the mean BMI was 24.34 ± 3.25 kg/m^2^, of which overweight (25 kg/m^2^ ≤ BMI < 28 kg/m^2^) patients accounted for 28.23% and patients with obesity (BMI ≥ 28 kg/m^2^) accounted for 10.48% [30]. The mean systolic blood pressure was 133.63 ± 18.67 mmHg, and the mean diastolic blood pressure was 82.05 ± 10.32 mmHg. The results are shown in Table 1.

The average follow-up duration of these patients managed by DMSCC was 0.98 (0.56, 1.30) years. Follow-up duration refers to the interval between the baseline and the time when the patient completes all the above questionnaires and HbA1c measurements at a follow-up after DMSCC management.

During the study period, the HbA1c levels of 46 (37.10%) patients exceeded the control standard by more than 1%. Among them, the dose intake of hypoglycemic drugs in 34 patients was appropriately increased, and the other 12 patients strengthened their diet and exercise management after the consultation of endocrinologists. A total of 13 (10.48%) patients had hypoglycemia once, and another 9 (7.26%) patients had hypoglycemia twice. All hypoglycemia were between <70 mg/dL (3.9 mmol/L) and ≥54 mg/dL (3.0 mmol/L). No severe hypoglycemia occurred.

### 3.2. Changes in HbA1c before and after Management

HbA1c is an important indicator and risk marker for assessing the level of glycemic control, self-management level, and treatment effect, which can reflect mean glycemia in the past 2-3 months. The ADA has set HbA1c < 7% (53 mmol/mol) as the target level for most diabetes patients to be well controlled [31]. In fact, the target value of HbA1c in clinical treatment is individualized. The target of HbA1c control in patients with older age, higher risk of hypoglycemia, shorter life expectancy, and severe complications or comorbidities may be appropriately relaxed [32].

Therefore, different HbA1c target values were set according to the age of the patients in this study. When the patient's age was <65 years old or ≥65 years old, HbA1c < 7% (53 mmol/mol) and HbA1c < 7.5% (58 mmol/mol) were considered to meet the control criteria, respectively. HbA1c compliance rate = number of patients with HbA1c meeting the control criteria/total number of patients × 100%. Conversely, it is considered to be poorly controlled when HbA1c < 9% (75 mmol/mol). HbA1c adverse rate = number of patients with poor HbA1c control/total number of patients × 100%.

The outcomes of our study showed that the average value of HbA1c was 7.92 ± 1.95% (63 ± 2 mmol/mol), the HbA1c compliance rate was 42.74%, and the HbA1c adverse rate was 25.81% at baseline before diabetes management.

After management of DMSCC, the average value of HbA1c was reduced to 6.94 ± 1.41% (52 ± 8 mmol/mol) (*t* = 6.23, *P* < 0.01), and the range of decrease in HbA1c was 0.98 ± 1.7%. Moreover, the HbA1c compliance rate was 69.35%, which was obviously improved compared to the premanagement level (*χ*^2^ = 25.56, *P* < 0.01), and the HbA1c adverse rate was decreased to 10.18% (*χ*^2^ = 15.70, *P* < 0.01). The differences were all statistically significant. The results are shown in Table 2.

### 3.3. Changes in Diabetes Self-Management Behavior before and after Management

Table 3 shows a comparative analysis of the SDSCA-6 scores before and after the diabetes management of the DMSCC. After comprehensive management and guidance in shared care clinic, patients with T2DM improved significantly in terms of “participating in physical activity for at least 30 minutes” (*S* = −402, *P* < 0.01), “testing blood glucose” (*S* = −847, *P* < 0.01), “testing your blood sugar the number of times recommended by your healthcare professional” (*S* = −1086, *P* < 0.01), “taking all your diabetes medications exactly as agreed with your healthcare professional” (*S* = −91, *P* < 0.05), and daily number of cigarettes (*S* = 36.5, *P* < 0.01) but not in terms of “following a certain healthy eating plan” (*S* = −216, *P* > 0.05) and “checking feet” (*S* = 269.5, *P* > 0.05).

### 3.4. Changes in Empowerment of Patients with Diabetes before and after Management

After diabetes management in the DMSCC, changes in the DES-DSF scores were mainly reflected in patients' significant improvement in terms of “trying different ways to treat your diabetes more effectively” (*S* = −356.5, *P* < 0.05). However, there is no significant difference in the other four aspects such as “telling others how they can help you better manage your diabetes” (*S* = −312, *P* > 0.05), “asking for support to help manage your diabetes when you need it” (*S* = −291, *P* > 0.05), “finding the information you need to treat your diabetes on your own” (*S* = −74.5, *P* > 0.05), and “participating in community activities to improve care for people with diabetes” (*S* = 52.5, *P* > 0.05). The results are shown in Table 4.

### 3.5. Changes in Psychological Distress of Patients with Diabetes before and after Management

Table 5 reflects the comparison of the PAID-5 scores before and after the diabetes management of the DMSCC. After the management, the psychological distress of the patients with diabetes was significantly improved. The changes are mainly reflected in terms of “feeling frightened when you think you have diabetes” (*S* = 856.5, *P* < 0.01), “feeling depressed when you think you have diabetes” (*S* = 895, *P* < 0.01), “concerned about possible serious complications of diabetes in the future” (*S* = 745.5, *P* < 0.01), “feeling that diabetes consumes too much of your energy and physical strength every day” (*S* = 637.5, *P* < 0.01), and “coping with complications of diabetes” (*S* = 555, *P* < 0.01).

## 4. Discussion

Through this study, we used a variety of scale tools to observed that the DMSCC can help patients have a positive impact on diabetes self-management behavior, lifestyle-related motivation and empowering ability, and diabetes-related emotional distress through a series of educational methods such as face-to-face and online consultation, assessment, guidance, and reminders. Moreover, the level of HbA1c and HbA1c compliance rate of patients with diabetes were significantly improved after shared care clinic management, which may have benefited from the above improvements.

In the management of chronic diseases, effective strategies for improving outcomes are often attributed to the following five areas: the use of a protocol, improved patient education, reorganization of practice systems and provider roles, greater availability of clinical information, and increased access to expertise. Continuous improvement in the above five aspects will help to form an integrated management and care system for chronic diseases [33]. As one of the most common chronic diseases, T2DM may lead to the development of complications, disability, and shorten life expectancy if not intervened in time, resulting in huge psychological, economic, and social burdens [1]. Therefore, it is required that the chronic disease management model needs to further optimize the process and details of diabetes education and management, and form a comprehensive and integrated management centered on diabetes patients, which has become an important guarantee for achieving comprehensive goals and high quality of life. This structure and goals are consistent with the DMSCC we conduct. Relatively speaking, regular diabetes care mainly focuses on diet, exercise, and insulin injection guidance, which is more generalized and limited, and is more often used for primary care or basic specialized care. Previous studies have confirmed that team care with the participation of specialist doctors can improve blood glucose better than regular care, and have a positive impact on midterm and long-term results [34]. As a form of team-based care, DMSCC has the advantage of being more comprehensive and meticulous [35, 36]. For example, the diet and exercise guidance are more systematic and can be continuously improved until the patient really learns to operate. Through the combination of online continuous care and regular outpatient follow-up, we can also achieve better and faster response in blood glucose monitoring, high and low blood glucose early warning, pharmaceutical guidance, complication management, problem solving, accompanying support, and other aspects, which is closer to the requirements of DSMES. To some extent, it may also be better than the simple offline team-based care [37]. In this study, it was indeed verified that when managed in a shared care clinic, patients with T2DM showed improvements in glycemic control and self-management abilities such as self-management behaviors, empowerment, and psychological distress.

Glycemic control is the foundation of treatment goals. For T2DM, one year with HbA1c > 7.5% (58 mmol/mol) loses around 100 life days [38]. Researches have shown that participation in DSMES can lead to significant reductions in HbA1c levels [24, 39–41]. The patients of T2DM in this study received nearly 1% reduction in HbA1c after receiving diabetes management of shared care clinic, which is consistent with the data from previous studies of HbA1c changes after receiving DSMES [24]. In China, the Endocrinology Department of Peking University First Hospital [42] and the Tianjin Medical University Zhu Xianyi Memorial Hospital [43] have also conducted the shared care clinic or unified care similar to this study, and their effects on reducing HbA1c and improving the HbA1c compliance rate has also been confirmed. It is worth noting that HbA1c and its compliance rate in patients with T2DM decreased significantly with the duration of diabetes [42, 44]. Therefore, diabetes education must be continuous, individualized, and change with the change of disease state.

In terms of diabetes self-management behavior changes, through the SDSCA-6 scale assessment, it was found that patients of T2DM had different levels of improvement in monitoring, being active, taking medication, and smoking restriction to reduce risk after diabetes management of shared care clinic. Increased diabetes knowledge, higher levels of health literacy, and higher social support were associated with better glycemic control and self-management behaviors in T2DM patients. Self-management behaviors are positively correlated with self-efficacy. Increased self-efficacy also contributes to lower HbA1c and enhancing well-being [4, 45, 46]. The Diabetes, Attitudes, Wishes, and Needs Second Study (DAWN2) is a global research project showing the current status of self-management and psychosocial support for people with diabetes in multiple countries. Overall, it was common for patients with diabetes to be able to follow medication and dietary recommendations but poorer in blood glucose monitoring, physical activity, and foot examination. Chinese patients outperformed patients in other countries in terms of healthy diet, physical activity, and treatment adherence, but were inferior in checking feet [26]. In the current research, the shared care clinic provided a multidisciplinary and individualized access to diabetes knowledge and basic medical and social support, and the app “Shared Care” reminded patients to monitor blood sugar and take medicines regularly and continuously and followed up on feedback on issues such as diet, exercise, and daily care. The results of our study showed that this approach can effectively promote the nonadvantage characteristics of blood glucose monitoring and consolidate the advantages of taking medication as required and enhancing physical activity. However, there was no significant improvement in adhering to a diet plan, which may be related to the higher basic dietary literacy of Chinese patients or the need to provide more popular and feasible dietary advice. There was no significant change in the patient's foot examination, which may be related to the lack of serious complications in the enrolled patients, and the lack of awareness of complication screening and prevention. Overall, helping patients understand the importance of achieving comprehensive diabetes treatment goals and actually do it may be one of the reasons for the patient's behavioral changes.

Empowerment is one of the critical factors influencing the self-care behaviors of patients with diabetes. The essence of empowerment is that the patients with diabetes assume full responsibility for self-management and making choices and actions, while the responsibility of the medical professional is to provide information, technology, and support to the patient. Self-efficacy and health literacy, such as literacy level and availability of support affects the empowerment [26, 47, 48]. Patients with different health literacy levels have different abilities to acquire, process, and understand diabetes education and management knowledge. Patients with low health literacy may have misconceptions or passive attitudes about diabetes management that make it difficult to seek or obtain details. Conversely, patients with high health literacy expect systematic, in-depth, and individualized counseling on lifestyle changes and medication [48]. Through the DMSCC in this study, patients with T2DM became willing to try different ways to treat diabetes more effectively. It may be related to access to diabetes knowledge, improved health literacy, and increased social support [49]. However, there was no significant improvement in patients' performance in actively seeking help for diabetes treatment, informing about their illness condition, and participating in community or peer activities.

The reduction of diabetes-related emotional distress and the implementation of diabetes self-management mutually reinforce each other [4, 23]. Appropriate psychological interventions can adjust knowledge, beliefs, and related cognitive structures, reduce emotional distress, improve well-being, and learn behavioral skills and coping [50]. Diabetes-related distress is associated with poor self-management and is more likely to lead to worse blood sugar levels and more complications [4, 23, 51]. Chinese healthcare providers pay less attention to their patients' mental health. Only about a quarter of patients were asked about their anxiety or depression, far less than the global average. Lack of psychological attention may also be one of the reasons why Chinese patients with diabetes have anxiety and depression, and do not know how to deal with them, resulting in more psychological burdens [52]. After diabetes education and management, the patient's diabetes-related distress and psychological problems can be improved [46, 53, 54]. As we managed patients in the DMSCC, patients' fear, depression, exhaustion about diabetes, concerns about complications, and inadequate coping were significantly improved.

Some limitations need to be considered. Because we are single-center research data, it may not be representative. In fact, some hospitals in China have conducted such DMSCC, and we are interested in data joint sharing. Another limitation is the limited length of the follow-up period. It is difficult to identify clinically significant differences during the follow-up within about one year, so it is planned to increase the follow-up period of the study, which will help the sustainability of the study. Regretfully, this article has not set up a regular care control group to better reflect the advantages of DMSCC. We have also started to collect data from the regular care group, but the quantity is small. So, it still needs further improvement. We are also looking forward to more rigorous and complete comparative data.

## 5. Conclusion

Chinese patients with T2DM have better empowerment ability, and are not inferior to the global level in terms of self-management behaviors [52]. DMSCC provides individualized prescriptions of hypoglycemic drugs, diet, exercise, medication, nursing guidance, etc., for patients to use. Most patients can follow the prescription to varying degrees under continuous in- and out-of-hospital, offline-online follow-up. We should continue to encourage empowerment and patient self-care and provide the multidimensional support and comprehensive guidance that patients need. The content and implementation of diabetes self-management education provided by the shared care clinics also needs to be adjusted according to the patient's disease process, psychological state, and health literacy level to achieve better outcomes. The DMSCC of our hospital can closely observe the patient's self-management attitude, ability, behaviors, and changes inside and outside the hospital by integrating multidisciplinary professional resources and taking advantage of new technologies. These positive changes in HbA1c compliance and diabetes self-management abilities such as self-management intentions, behaviors, empowerment, and diabetes-related emotional distress in these patients with T2DM give us confidence that this work is meaningful and patient beneficial as an effective exploration and practice of DSMES. Shared clinic care deserves to be extended to almost all patients with diabetes. Patients with different courses of disease, types of complications, or concerns may find their own key points, obtain support, and make some progress in different aspects of diabetes management.

## Figures and Tables

**Table 1:** Demographic and clinical characteristics at baseline.

Characteristics	Baseline data of total enrolled patients (*n* = 124)
Gender	
Male	76 (61.29%)
Female	48 (38.51%)
Age (years)	54.57 ± 12.23
Marital status	
Married	118 (95.16%)
Unmarried	6 (4.84%)
Highest level of education	
Primary school	27 (21.77%)
Junior high school	49 (39.52%)
Senior high school	32 (25.81%)
Bachelor degree or above	16 (12.90%)
Smoking status	
Smoking	20 (16.13%)
No smoking	104 (83.87%)
Duration of T2DM (years)	6.85 ± 7.21
BMI (kg/m^2^)	24.34 ± 3.25
Overweight	35 (28.23%)
Obesity	13 (10.48%)
SBP (mmHg)	133.63 ± 18.67
DBP (mmHg)	82.05 ± 10.32
Application of glucose-lowering agents	
No glucose-lowering agents	20 (16.13%)
Application of OADs^a^	97 (78.23%)
One type of OADs	29 (23.39%)
Two types of OADs	49 (39.52%)
Three types of OADs	18 (14.52%)
Four types of OADs	1 (0.81%)
Application of insulin	48 (38.71%)
One type of insulins	42 (33.87%)
Two types of insulins	6 (4.84%)
Application of GLP-1RA	3 (2.42%)
Scheme of glucose-lowering agents	
OAD(s) only	53 (42.74%)
Insulin only	7 (5.65%)
OAD(s) + insulin	41 (33.06%)
OAD(s) + GLP-1RA	3 (2.42%)

*Note*. ^a^OADs include biguanides, sulfonylureas, meglitinides (glinides), dipeptidyl peptidase 4 (DPP-4) inhibitors, sodium-glucose cotransporter 2 (SGLT2) inhibitors, thiazolidinediones (TZDs), and *α*-glucosidase inhibitors. ^b^Insulins include rapid-acting, short-acting, intermediate-acting (NPH), long-acting, and premixed insulin. OADs: oral antidiabetic drugs. GLP-1RA: glucagon-like peptide 1 receptor agonist.

**Table 2:** Comparison of HbA1c and HbA1c compliance before and after management of DMSC**C**.

	Baseline	After management	*t* value or *χ*^2^value	*P* value
HbA1c, % (mmol/mol)	7.92 ± 1.95 (63 ± 2)	6.94 ± 1.41 (52 ± 8)	6.23^c^	<0.0001
HbA1c compliance rate, %^a^	42.74%	69.35%	25.56^d^	<0.0001
HbA1c adverse rate, %^b^	25.81%	10.18%	15.70^d^	<0.0001

*Note*. ^a^HbA1c compliance rate = number of patients with HbA1c meeting the control criteria (<65 years old: HbA1c < 7% (53 mmol/mol) or ≥65 years old: HbA1c < 7.5% (58 mmol/mol))/total number of patients × 100%. ^b^HbA1c adverse rate = number of patients with poor HbA1c control (HbA1c > 9% (75 mmol/mol))/total number of patients × 100%. ^c^The *t* value after the paired-sample *t*-test. ^d^The *χ*^2^ value of Fisher's exact probability test followed by paired chi-square test (McNemar's test).

**Table 3:** Comparison of SDSCA-6 scores before and after management of DMSC**C**.

Question^a^	Baseline M (IQR)	After management M (IQR)	*S* value^b^	*P* value
Q1. Have you followed a healthy eating plan? (e.g., eat in moderation, eat less high-fat or high-sugar foods)?	5 (3)	6 (4)	−216	0.3089
Q2. Did you participate in physical activity for at least 30 minutes?	7 (3)	7 (2)	−402	0.0022
Q3. Did you test your blood glucose?	2 (2.5)	3 (5)	−847	<0.0001
Q4. Did you test your blood sugar the number of times recommended by your healthcare professional?	1 (3)	3 (5)	−1086	<0.0001
Q5. Did you check your feet?	1 (5)	0 (3)	269.5	0.1381
Q6. Did you take all your diabetes medications exactly as agreed with your healthcare professional?	7 (0)	7 (0)	−91	0.0193
Q7. Your smoking condition: no/yes (if yes, fill in the average number of cigarettes smoked per day)	0 (0)	0 (0)	36.5	0.0085

*Note*. ^a^The answers of “0–7” for Q1–Q6 represent 0–7 days in the last week that match the description of the problem. The number filled in Q7 is the average number of cigarettes smoked per day in the past week. ^b^The *S* value after paired-sample Wilcoxon signed-rank test. M (IQR): median (interquartile range).

**Table 4:** Comparison of DES-DSF scores before and after management of DMSCC.

Question^a^	Baseline M (IQR)	After management M (IQR)	*S* value^b^	*P* value
Q1. Tell others how they can help you better manage your diabetes	1 (1.5)	1 (2)	−312	0.0634
Q2. Try different ways to treat your diabetes more effectively	1 (1)	1 (2)	−356.5	0.0287
Q3. Ask for support to help manage your diabetes when you need it	1 (1.5)	1 (2)	−291	0.0770
Q4. Find the information you need to treat your diabetes on your own	2 (2)	2 (2)	−74.5	0.6398
Q5. Participate in community activities to improve care for people with diabetes	1 (1)	1 (0)	52.5	0.5809

*Note*. ^a^The answers of “1–5” for Q1–Q5 represent as follows: 1 point: never, 2 points: rarely, 3 points: sometimes, 4 points: often, and 5 points: always. ^b^The *S* value after paired-sample Wilcoxon signed-rank test. M (IQR): median (interquartile range).

**Table 5:** Comparison of PAID-5 score before and after management of DMSCC.

Question^a^	Baseline M (IQR)	After management M (IQR)	*S* value^b^	*P* value
Q1. You are frightened when you think you have diabetes	1 (1)	0 (1)	856.5	<0.0001
Q2. You feel depressed when you think you have diabetes	1 (1)	0 (1)	895	<0.0001
Q3. Concerned about possible serious complications of diabetes in the future	1 (1)	0 (1)	745.5	<0.0001
Q4. It feels like diabetes consumes too much of your energy and physical strength every day	1 (1)	0 (1)	637.5	<0.0001
Q5. Coping with complications of diabetes	1 (1)	0 (1)	555	<0.0001

*Note*. ^a^The answers of “0–4” for Q1–Q5 represent as follows: 0 point: no problem, 1 point: minor problems, 2 points: moderate problems, 3 points: some serious problems, and 4 points: serious problems. ^b^The *S* value after paired-sample Wilcoxon signed-rank test. M (IQR): median (interquartile range).

## Data Availability

The datasets generated and/or analyzed during the current study are not publicly available but are available from the corresponding author on reasonable request.
